# Gene Mining for Proline Based Signaling Proteins in Cell Wall of *Arabidopsis thaliana*

**DOI:** 10.3389/fpls.2017.00233

**Published:** 2017-02-27

**Authors:** Muhammad Z. Ihsan, Samina J. N. Ahmad, Zahid Hussain Shah, Hafiz M. Rehman, Zubair Aslam, Ishita Ahuja, Atle M. Bones, Jam N. Ahmad

**Affiliations:** ^1^Cholistan Institute of Desert Studies, The Islamia University BahawalpurBahawalpur, Pakistan; ^2^Plant Stress Physiology and Molecular Biology Lab, Department of Botany, University of Agriculture FaisalabadFaisalabad, Pakistan; ^3^Integrated Genomics Cellular Developmental and Biotechnology Lab, Department of Entomology, University of Agriculture FaisalabadFaisalabad, Pakistan; ^4^Department of Arid Land Agriculture, Faculty of Meteorology, King Abdulaziz UniversityJeddah, Saudi Arabia; ^5^Department of Electronic and Biomedical Engineering, Chonnam National UniversityGwangju, South Korea; ^6^Department of Agronomy, University of Agriculture FaisalabadFaisalabad, Pakistan; ^7^Department of Biology, Norwegian University of Science and TechnologyTrondheim, Norway

**Keywords:** *Arabidopsis*, co-expression, geneMANIA, GENEVESTIGATOR, kinase, proline

## Abstract

The cell wall (CW) as a first line of defense against biotic and abiotic stresses is of primary importance in plant biology. The proteins associated with cell walls play a significant role in determining a plant's sustainability to adverse environmental conditions. In this work, the genes encoding cell wall proteins (CWPs) in Arabidopsis were identified and functionally classified using geneMANIA and GENEVESTIGATOR with published microarrays data. This yielded 1605 genes, out of which 58 genes encoded proline-rich proteins (PRPs) and glycine-rich proteins (GRPs). Here, we have focused on the cellular compartmentalization, biological processes, and molecular functioning of proline-rich CWPs along with their expression at different plant developmental stages. The mined genes were categorized into five classes on the basis of the type of PRPs encoded in the cell wall of *Arabidopsis thaliana*. We review the domain structure and function of each class of protein, many with respect to the developmental stages of the plant. We have then used networks, hierarchical clustering and correlations to analyze co-expression, co-localization, genetic, and physical interactions and shared protein domains of these PRPs. This has given us further insight into these functionally important CWPs and identified a number of potentially new cell-wall related proteins in *A. thaliana*.

## The plant cell wall

The cell wall (CW), considered as first line of defense in plants, is composed of polysaccharides (cellulose, hemicellulose, pectin), and proteins. These proteins can either be structural or non-structural depending upon their functionality. Since the first report of cell wall proteins (CWPs) in *Hydrodictyon africanumin* (Northcote et al., 1960), hundreds of proteins have been identified which serve as an integral structural part (about 10% of wall dry weight) and perform multiple functions in various signaling pathways.

**Figure 1:**
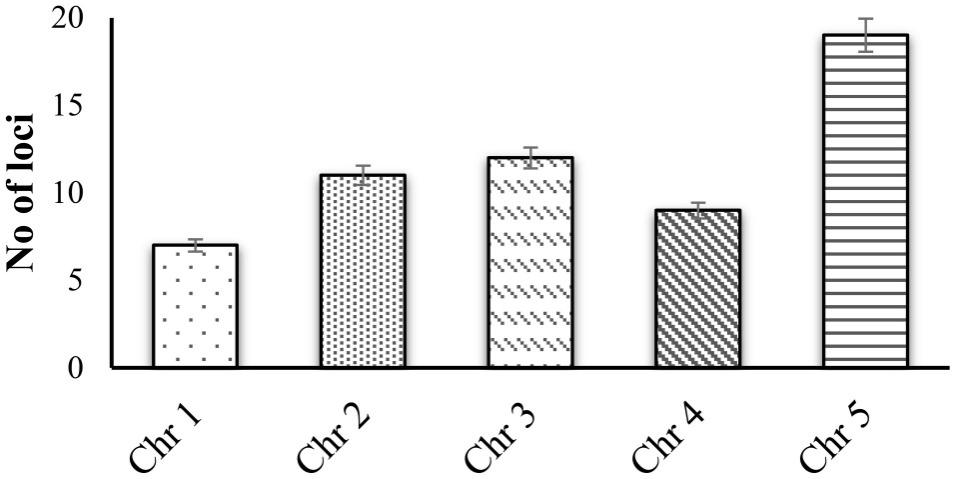
**Gene mining approach for identification genes that are involved in proline based regulation in CW of *Arabidopsis thaliana***.

**Figure 2:**
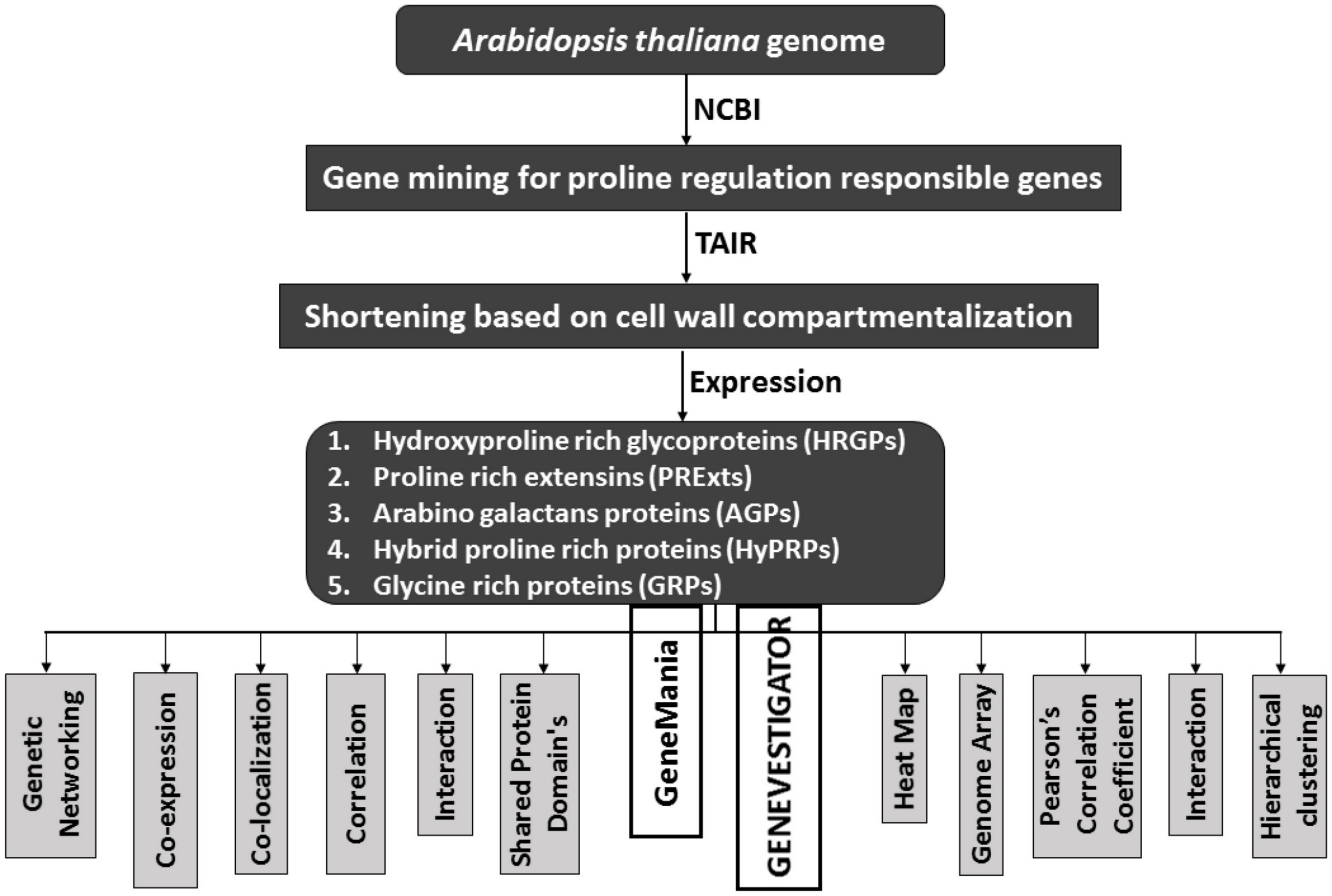
**A schematic diagram to show the flow of proline based genes regulation in CW of *Arabidopsis thaliana***.

**Figure 3:**
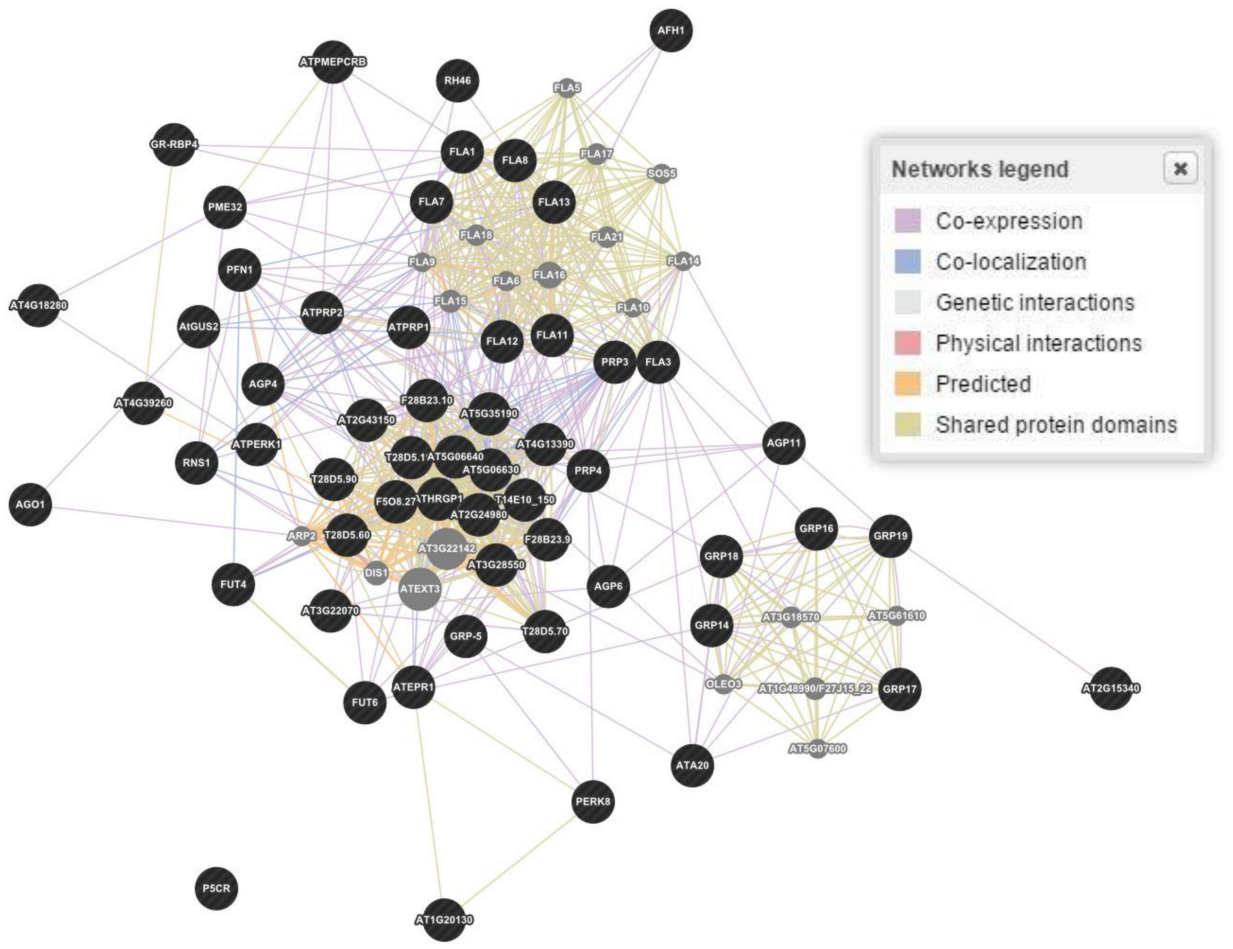
**Gene and interaction networks for molecular functioning of genes involved in regulation of proline based cell wall proteins in *Arabidopsis thaliana***. Physical interactions 55.7, 7.4, and 39.0%; Predicted interaction 17.5, 30.7, and 30.8%; Co-expression 15.6, 55.9, and 11.8%; Genetic interactions 6.9, 1.1, and 3.3%; Shared protein domains 3.6, 2.9, and 14.6%; Co-localization 0.6, 1.8, and 0.4% were estimated for genes biological processes, cellular components and molecular functions, respectively. Figure represents molecular functions networks as a reference. Dark (black) spots highlight genes interacting for a specific family of proteins, while light (gray) spots represent those genes whose interactions were not considered.

**Figure 4:**
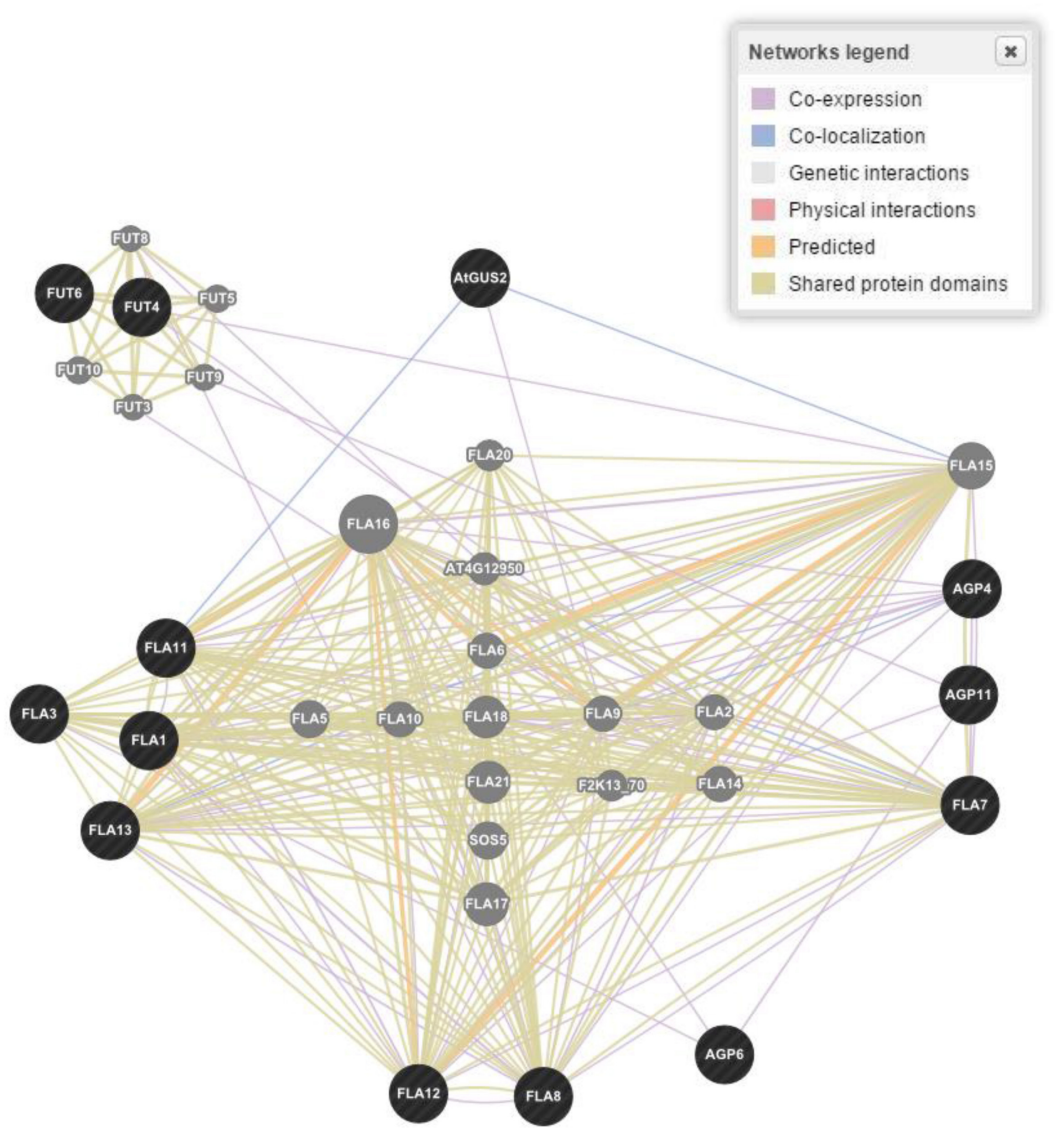
**Gene networking and interactions for one of the four responsible gene families based on biological process, molecular functions and cellular components in *Arabidopsis thaliana***. Figure represents only proline rich proteins class as a reference. Dark (black) spots highlight genes interacting for a specific family of proteins, while light (gray) spots represent those genes whose interactions were not considered.

**Figure 5:**
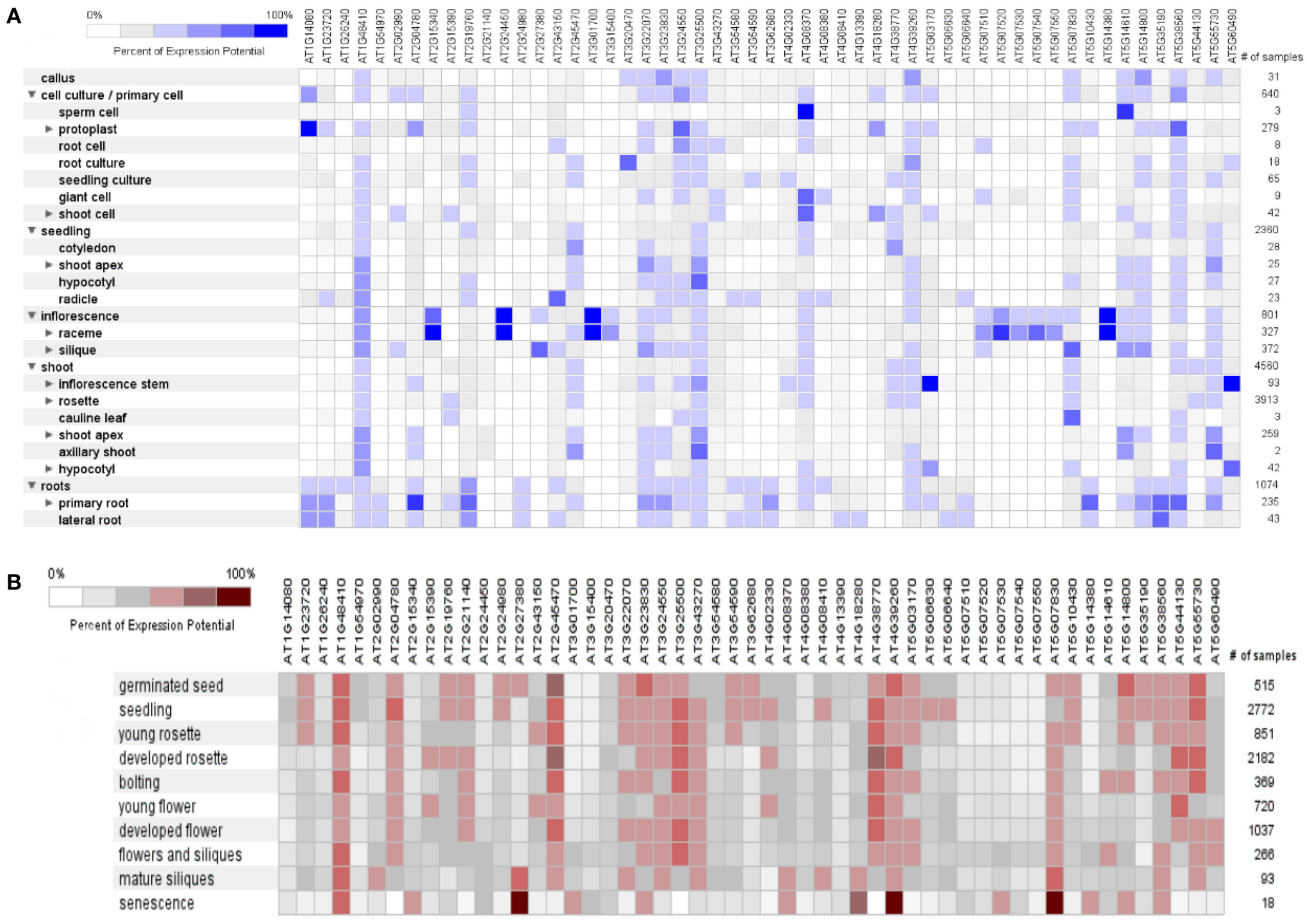
**A meta-analysis approach representing heat maps of proline responsible genes in *Arabidopsis thaliana***. **(A)** Expression potential (%) in anatomical plant parts, **(B)** Expression potential (%) in different developmental stages (from germination to senescence). White color represents 0% percent expression level while dark blue color represents 100% level of expression. Change in each level represents 20% increase or decrease in expression potential of each gene at different developmental stages.

**Figure 6:**
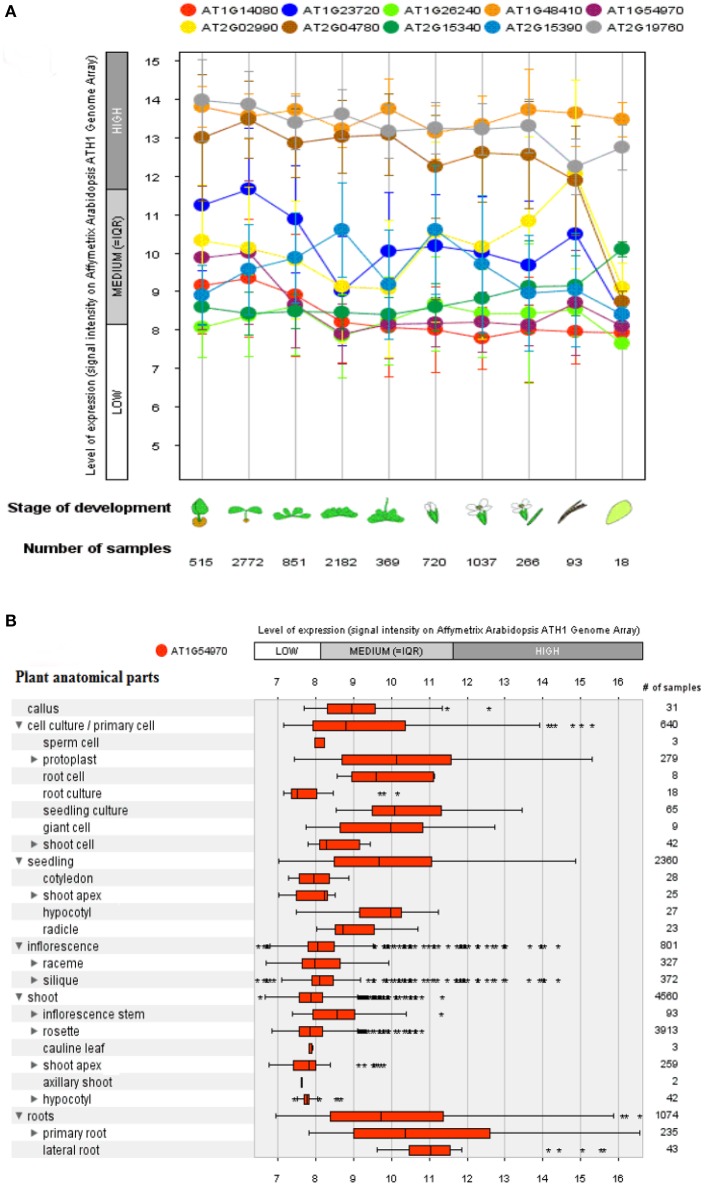
**A genome array map representing levels of expression of 10 selected genes at different developmental stages and a single selected gene for different anatomical parts in *Arabidopsis thaliana***. **(A)** Levels of expression at different developmental stages (from germination to senescence), which has been analyzed against different number of samples. **(B)** Expression level of a single selected gene AT1G54970 (*PRP1*) in 27 anatomical parts. Bars represent standard error at *P* ≤ 0.05.

**Figure 7:**
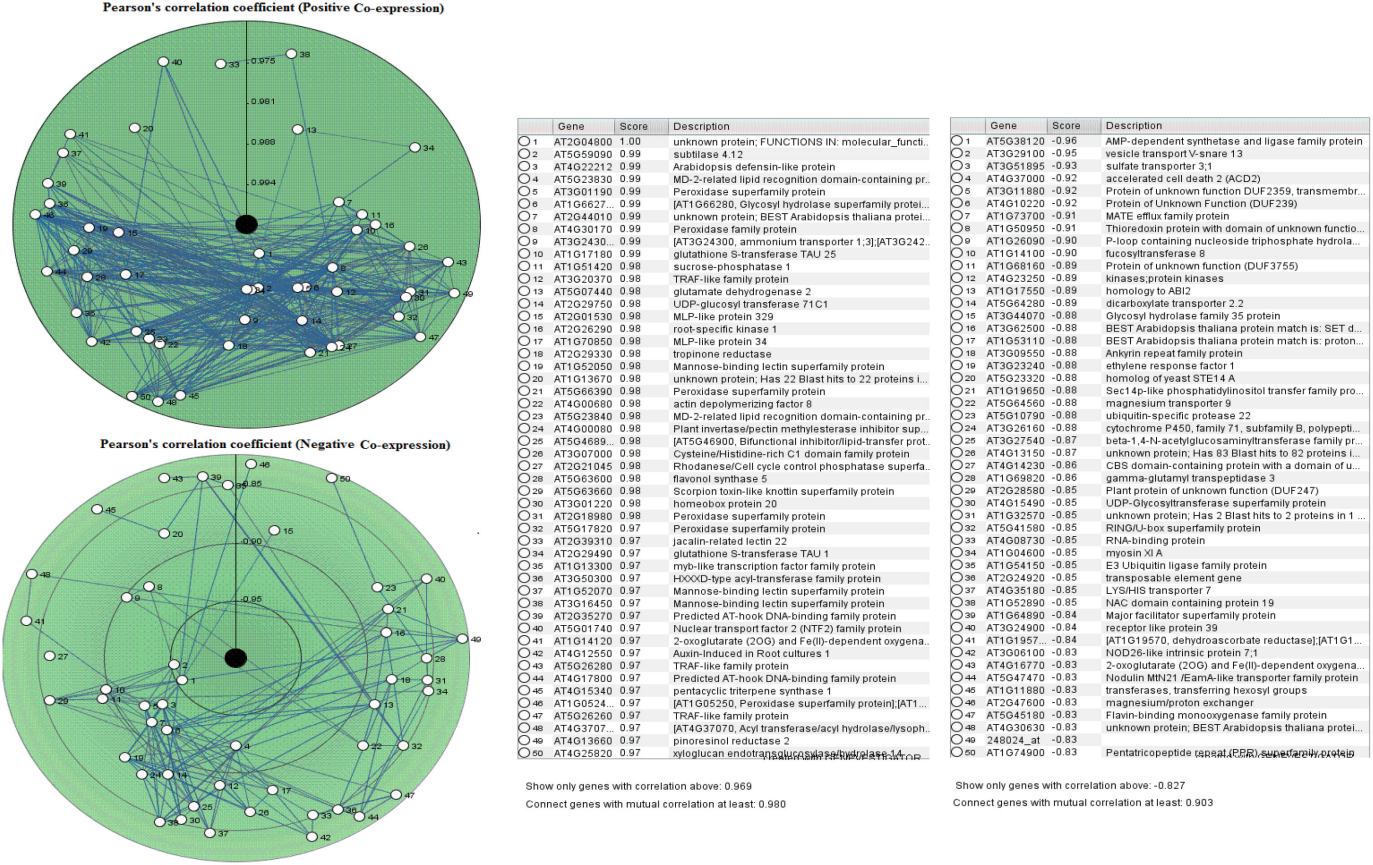
**Pearson's correlation coefficient for proline based CW regulating selected genes in *Arabidopsis thaliana* for positive and negative co-expressions, respectively**.

**Figure 8:**
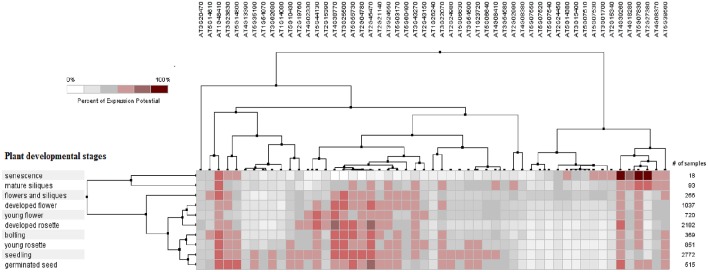
**Pearson's based hierarchical clustering with percent of expression potential for proline based CW compartmentalized genes of *Arabidopsis thaliana* across 10 different developmental stages**. White color represents 0% percent level of expression while dark red color.

CWPs have key importance in sensing environmental stresses and controlling CW dynamics in response to the growth and development of the plant. However, currently we have a limited understanding of the structure, function and interaction of CWPs, and also very little knowledge of association of cuticle with plants reactive phytochemicals (Ahuja et al., 2016). Proline rich proteins (PRPs), proline rich extensin like proteins (PRExts), hydroxy-proline rich O-glycoproteins (HRGPs), expansins, and formin like proteins are some of the known classes of CW proteins with covalent scaffold and glycosylation as their known interactions (Boron et al., 2014; Suzuki et al., 2015). *Arabidopsis thaliana* is a model plant comprising of five chromosomes with 33,542 genes, where 1,605 genes are responsible for the CW development. Out of these 1,605 genes, 252 are responsible for cellulose, 10 for hemicellulose, and 317 for pectin regulation (Albenne et al., 2013). In this review, we have mainly described PRPs, which are the pivotal constituent of the CW together with mining of genes behind these proteins. Along with this, we have attempted to build wired networks to see how these genes co-express and interact in regulating PRPs during the biological and physical processes as well as in determining the molecular functions. We have used the gene mining approach to identify 58 genes located on different chromosomes (Figure 1). These genes are either directly or indirectly involved in the regulation of proline related proteins in the CW under various biotic and abiotic stresses. We have classified these genes into five groups based on their expression for the different structural and functional proteins (Figure 2). Moreover, schematic diagrams (using GeneMANIA and GENEVESTIGATOR) have been generated for the networks of gene co-expression, gene co-localization, genetic interaction, physical interaction, shared protein domains, and predicted interaction (Figures 2–**8**). A heat map, genome array, Pearson's correlation coefficient (PCC) and hierarchical clustering are also presented for the selected genes for an estimation of the genetic interactions and their level of co-expressions at different plant developmental stages and in the various plant anatomical parts.

## Cell wall proteins (CWPs)

The CWPs are divided into nine classes based on their signaling events (Albenne et al., 2013). They are linked to the several important pathways including lipid and carbohydrate metabolism, structural components, proteolytic andoxidoreductive activity, cell signaling, molecular interaction, miscellaneous, and the proteins with an unknown activity.

The CWPs involved in cell signaling, in response to abiotic stresses, have been extensively studied. Under such stress conditions, the major classification of CWPs include the salt overly sensitive kinases, phospholipases, transcription factors, dehydration responsive element binding proteins, C-repeat binding factor, mitogen activated proteins, and abscisic acid responsive element binding factors (Vinocur and Altman, 2005). The involvement of CW in different stress reception mechanisms is not surprising. Kinases are perceived as potential candidates for the CW sensor (Steinwand and Kieber, 2010). Activation of various kinases in response to the changing levels of the same stress has already been well-reported (Kacperska, 2004). In *A. thaliana*, 26 genes related to the CW associated kinases (CWAKs), and similar functions have been reported (Verica and He, 2002). In addition to the abiotic stresses, CWAKs are also involved in the plant defense against pathogens (Bellincampi et al., 2014). Recently, a number of new CWAKs have been reported, which include proline rich extensin like receptor kinases (PERKs), leucine rich repeats receptor like kinases (LRKs), and lectin receptor kinases (LecRKs) (Wolf et al., 2012). The CW plasma membrane interface is hypothesized as a key site for the stress signal perception where the interaction was studied between arabinogalactan proteins (AGPs) and receptor like kinases (Baluška et al., 2003). The production of hydrogen peroxide and downward redox signaling during the stress is an interesting aspect of CWPs (Spasojević and Pristov, 2010). The generation of reactive oxygen species (ROS) in response to CWP signaling (Barceló and Laura, 2009) is an important and interesting phenomenon, because mitochondria and chloroplast are considered as the major players of ROS production (Voothuluru and Sharp, 2013). In response to abiotic stresses, the extracellular ROS accumulation is tightly regulated by the enzymes (Jaspers and Kangasjärvi, 2010) in cell membrane, which in turn are tightly bonded to the CW (Plieth, 2012). The speedy response of the CW (associated with the changes in its composition or structure) has led researchers to make a detailed study of the various functional proteins with an enzymatic activity within the CW. These include the CW formation, reorganization, loosening and carbohydrate metabolism (Brown et al., 2005; Gupta et al., 2005; Sasidharan et al., 2011; Xu et al., 2014).

Collectively, 2,170 CWPs have been identified on the basis of their distinct gene expression in various plants (San Clemente and Jamet, 2015). The glycoside hydrolases, lyases, esterases, and hydrolases come under the umbrella of proteins acting on polysaccharides (Jamet et al., 2008). The other class of oxidoreductases contains blue copper binding proteins, multi-copper oxidases and peroxidases, while the proteases consist of Aspartate (Asp) proteases, ser carboxy peptidases, and cysteine (Cys) proteases. The oxidoreductases are an important class of enzymes that transfer OH group at critical physiological stages of plant development and affect the structure of the CW (Fry, 1998). Lipid transfer proteins are involved in lipid metabolism (Lev, 2010), and the AGPs in stress signaling (Shen et al., 2001). While, extensins (EXTs) and glycine rich proteins (GRPs) are the structural proteins. Some proteins, grouped as miscellaneous class like germin and germin like proteins, phosphatases and the phosphate inducible proteins, are still unclassified (Shahzad et al., 2013).

The *in silico* analyses showed 58 genes responsible for the regulation of proline based proteins in the CW of *A. thaliana* (Figure 3). The gene interaction and co-expression in the form of a wired network has been constructed for the cellular component and biological and molecular process. A significant variability has been observed in the degree of physical interactions, predicted interactions, co-expressions, genetic interactions, shared protein domains, and co-localization.

### Hydroxy-proline rich O-glycoproteins (HRGPs)

The HRGPs were recognized several decades earlier than the CWPs and marked as the complex macromolecules based on their chemistry and functionality (Wang et al., 2012). Based on glycosylation, HRGPs are categorized into three sub-classes. These classes are hyper-glycosylated AGPs, moderately glycosylated EXTs and hyper PRPs (Tan et al., 2004). The HRGPs showed a specific multitude of functionalities. The cell signaling, defense, embryogenesis, development, reproduction, and expression are some of the recognized functions of AGPs (Seifert and Roberts, 2007). The EXTs are involved in the covalent scaffold and portrayed as the structural proteins of the CW (Cannon et al., 2008). The PRPs are the least developed proteins, and linked with the numerous biotic and abiotic stresses (Battaglia et al., 2007). The diversity of HRGPs further enhanced the addition of hybrid and chimeric proteins into the HRGPs family (Showalter et al., 2010). The gene mining of *A. thaliana* has revealed that 166 genes are encoding HRGPs, whereas 85 genes encode AGPs, 59 genes EXTs, 18 genes PRPs, and 4 genes hybrid proteins (Showalter et al., 2010). More than 50 genes were identified on the basis of their involvement in proline regulation in the CW for the 15 different functions. Even after more than 60 years of research, the detailed expression and functioning of HRGPs has not been clarified (Léonard et al., 2010). Several classes of the proteins share common function and sometimes a single class in the CW controls more than one function (Jamet et al., 2006). The AGPs are considered as the signaling proteoglycans but also sometime implicated to link the CW to the plasma lemma (Ellis et al., 2010). The EXTs play a vital role in the CW architecture (Lamport et al., 2011).

The HRGPs are evidently involved in growth, development, embryogenesis, apoptosis, and the CW architecture (Tan et al., 2012). The AGPs can be further divided into several classes, which may belong to the classical AGPs, non-classical AGPs, AG peptides, Lys-rich AGPs, Fasciclin-like AGPs (FLAs), and chimeric AGPs (Schultz et al., 2002). The AGPs attached to the cell membranes (Gaspar et al., 2001), are encoded by 69 genes involved in the stress signaling and cellular processes (Ma and Zhao, 2010). The EXTs, under the pathogenic attack, were engaged in the peroxidase mediated cross-link to reduce its permeability (Cannon et al., 2008). To face stress in a better way (Ihsan et al., 2016), plant cells accumulate osmolytes (hydro-soluble carbohydrates) and proline to combat a water loss (Yamaguchi and Blumwald, 2005). Proline is synthesized from glutamate via a two-step oxido-reductase pathway involving the pyrroline-5-carboxylate synthase (P5CS) γ-glutamyl kinase (γ-GK), and glutamic-γ-semialdehyde dehydrogenase (GSA-DH; Chen et al., 2009). Increase in proline in response to stress is associated with the upregulation of its biosynthetic genes (Silva-Ortega et al., 2008). Thus, both proline levels and the expression of P5CS are useful markers for assessing the levels of stress acclimation through modifications in structure of the CW. It has been reported that overexpression of a novel feedback-desensitized Δ1-pyrroline-5-carboxylate synthetase increased proline accumulation in transgenic *Nicotiana plumbaginifolia* thereby conferring the salt tolerance in this plant (Ahmed et al., 2015).

The wired networking of genes, constructed through GENEVESTIGATOR, revealed a high extent of interaction and co-expression of clusters of genes controlling these classes of proteins (Figure 4). Differential interaction and co-expression has been observed between the genes for biological processes, molecular functions and cellular compartmentalization.

### Proline rich extensin like proteins (PRExts)

The PRExts are characterized partially in the superfamily of HRGPs and are implied in the assemblage of the CW and promotion of the cell growth and shape (Sasidharan et al., 2011). They have been studied extensively in previous decades (de Caestecker et al., 2000; Silva and Goring, 2002; Hsu et al., 2005; Bai et al., 2009). They formulate a highly known CWPs family. These are basic pectin interacting proteins containing Hyp O-glycosylated with short arabino-oligosaccharides. They can configure a helical structure named polyproline II, cross-linked through isodityrosine or di-isodityrosine (Choe and Cosgrove, 2010). *In vitro* scrutiny of atomic force microscopy has explored the pure form of *A. thaliana* “*EXT3*” constituting branchy structures, consistently cross-linked by the peroxidases (Geilfus et al., 2010). Likewise, threonine-rich hydroxyproline-rich glycoprotein (THRGP) found in maize were not cross-allied by the peroxidases. It was anticipated that the positive charged scaffolds produced by the assembly of EXTs in cell plates of the cell wall positively react with charged pectin through an ionic force. The presence of covalent interactions has also been proposed between EXTs and pectin. It has been found that a three dimensional covalent network was formed by the EXTs via Tyrosine (Tyr) linkages mediated by EXT (Cannon et al., 2008). The EXT monomers assemble in the CW in terminal zipper like organization through a cross linkage (Lamport et al., 2011). It is projected that an EXT associated with pectin further blossoms cell wall through an acid base reaction by forming a supra-molecular ionic structure. The EXTs constitute a three dimensional network of the glycoproteins with a pectin component of the CW (Voragen et al., 2009). The occurrence of the EXTs like chimeras and hybrid EXTs have also been confirmed in the CW (Showalter et al., 2010). Despite of the EXTs insolubility, their behavior has also been modified by the other domains of the proteins.

The characteristics articulated by the EXTs were just like the collagen cross-linked forming motifs (Lodish et al., 1999). However, contrary to collagen, the EXTs exhibit a plant specific post translational feature named *O*-glycosylation on the Ser-Hyp motifs. The experimental methodologies opted through molecular dynamics and homology modeling, recommended that classical EXTs would form a triple helical structure via the lateral staggered configuration and a Tyr cross-linking analogous to the collagen (Cannon et al., 2008). In the genome of *A. thaliana*, EXT is mentioned in the form of 59 members, like classical, chimeras and hybrids occupied by the different domains. No doubt, high number of EXT domains are residing in the CW but a little is known about their exact functionality and diversity during the plant developmental stages (Lamport et al., 2011). The analogous and repetitive sequences of proteins, encoding of a large number of proteins in same genome and simultaneous expression of the genes in the same tissue of plant are the different grounds that had created difficulty for us to perceive the exact biology of the EXTs.

The O-glycoproteins possessing EXT domains were finally integrated in the CW, put together by the different post translational modifications (PTMs), comprising processing of signal peptide by endoplasmic reticulum, proline hydroxylation, O-glycosylation, and Tyr cross linking in the CW (Nguema-Ona et al., 2014). In the past few years, research has revealed that several enzymes were involved in EXT fabrication pathways as a part of their PTMs. Even a small change in O-glycosylation status of EXTs affected the expansion of the polarized cell as observed by a drastic root hair appearance in mutants in response to the absence of glycosyltransferase (Velasquez et al., 2011). It has been reported that both types of the *O*-glycosylation located in the EXTs, were needed for the correct functionality of the EXTs during the root elongation. Somehow, it is not certain how the EXT monomer assembled into glycol-network and how the EXT pectin interactions are regulated during the nascent CW formation (Micheli, 2001). Through bioinformatics tools, we have found 21 genes encoding the EXTs like proteins in the CW that determined its structural architect at the molecular level and were expressed in different parts of the plant (Table 1).

**Table 1:** **Proline-rich extensins genes in cell wall of *A. thaliana*^†^**.

**Locus**	**Gene ID**	**Biological process/Molecular function**	**Expressed/Located**	**Description**	**Some of related the references**
AT2G27380	*EPR1*	Component of CW	Micropylar endosperm	Extensin proline-rich 1	Penfield et al., 2006
AT3G24550	*PERK1*	Protein phosphorylation and ATP binding	Carpel, cauline leaf and cotyledon	Proline-rich extensin-like protein receptor kinase 1	Nemoto et al., 2011
AT4G08410		CW organization and CW structural constituent	Hypocotyl, plant embryo, root, sepal, and shoot apex	Proline-rich extensin-like protein	Velasquez et al., 2011
AT1G26250	*EXT18*	CW organization and CW structural constituent	Endomembrane system	Proline-rich extensin-like family protein	Renault et al., 2013
AT5G06640	*EXT10*	CW organization and CW structural constituent	Hypocotyl, root, root hair cell, sepal, shoot apex, trichoblast, and vascular leaf	Proline-rich extensin-like family protein	Bruex et al., 2012
AT2G43150		CW organization and CW structural constituent	Carpel, leaf structure, guard cell, hypocotyl, petal plant embryo, shoot apex, stem, and vascular leaf	Proline-rich extensin-like family protein	Sottosanto et al., 2004
AT4G08370		CW organization and CW structural constituent	Endomembrane system	Proline-rich extensin-like family protein	Armengaud et al., 2004
AT4G08400		CW organization and CW structural constituent	Pollen	Proline-rich extensin-like family protein	–
AT1G26240		CW organization and CW structural constituent	Root	Proline-rich extensin-like family protein	–
AT1G23720		CW organization and CW structural constituent	Carpel, hypocotyl, and root	Proline-rich extensin-like family protein	Zhu et al., 2006
AT3G54580		CW organization and CW structural constituent	Pollen, pollen tube, root, root hair cell, and trichoblast	Proline-rich extensin-like family protein	Bruex et al., 2012
AT3G28550		CW organization and CW structural constituent	Endomembrane system	Proline-rich extensin-like family protein	–
AT5G35190	*EXT13*	CW organization and CW structural constituent	Root, root hair cell, and trichoblast	Proline-rich extensin-like family protein	Ma and Bohnert, 2007
AT4G13390	*EXT12*	CW organization and CW structural constituent	Root hair cell and trichoblast	Proline-rich extensin-like family protein	Diet et al., 2006
AT2G24980	*EXT6*	CW organization and CW structural constituent	Root	Proline-rich extensin-like family protein	Velasquez et al., 2011
AT5G06630		CW organization and CW structural constituent	Collective leaf structure, hypocotyl, pollen, root, and vascular leaf	Proline-rich extensin-like family protein	Dinneny et al., 2008
AT1G20130		Lipid metabolic process, lipase activity and CW structural constituent	Extracellular region	GDSL-motif esterase/acyltransferase/lipase	Hanada et al., 2011
AT5G38560	*PERK8*	Protein phosphorylation, ATP binding, and kinase activity	Carpel, cauline leaf, collective leaf structure, cotyledon, and cultured plant cell	Proline-rich extensin-like protein receptor kinase 8	Humphrey et al., 2014
AT5G49080	*EXT11*	CW structural constituent	Root hair cell, synergid and trichoblast	Similar to proline-rich extensin-like family protein	Wuest et al., 2010
AT3G54590	*HRGP1*	CW structural constituent	Carpel, cotyledon, flower, hypocotyl, and inflorescence meristem	Hydroxyproline-rich glycoprotein	Wang et al., 2008
AT4G08380		–	Synergid	Proline-rich extensin-like family protein	Wuest et al., 2010

† *The information given in this table is based on TAIR database (Lamesch et al., 2012)*.

### Arabinogalactan proteins (AGPs)

The AGPs are proteoglycans found in nearly all tissues and exudates of higher plants (Youl et al., 1998). These are 90% polysaccharides by composition and can be extracted in a low salt buffer and have been reported as non-structural part of the CW matrix (Fincher et al., 1983). These proteins belonged to the highly diversified hydroxyl proline-rich glycoproteins superfamily (Velasquez et al., 2011) in the plant kingdom (Seifert and Roberts, 2007). In *Arabidopsis*, the AGPs have been classified into 22 classes on the basis of their proteoglycan formation cohering with various developmental processes in plants (Showalter et al., 2010). However, the pectin and cellulose form the network structured by AGPs (Jia et al., 2015), which maintains the structural integrity of the CW. Moreover, the higher plant CWs constituted by cellulose micro-fibrils in glycoproteins, pectin and cellulose maintained the functional features, integrity and strength of the CW (Schwager et al., 2007; Ellis et al., 2010). Various studies have confirmed the prevalence of association between AGPs and pectin's from plants tissue e.g., grapes, carrot and sugar beet. Co-localization of pectin's and AGPs has been observed in the pollen tube (Mollet et al., 2002). When a plant CW was treated with the pectin degrading enzymes, an increase in the release of AGPs was observed, confirming an association between AGPs and pectin (Lamport et al., 2006). The interactions have also been reported between the AGPs and polysaccharides such as AGP-xylan complexes (Keegstra et al., 1973; Kwan and Morvan, 1995). An isoform of *A. thaliana* AGP (At3g45230) has been shown to be covalently linked with the pectins and hemicelluloses (Tan et al., 2012). Some AGPs function as polysaccharide plasticizers as they establish a cross linkage in the CW (Lamport et al., 2006).

A few AGPs were exposed to make the covalent interactions with the CW implying its role as a cross linker and pectin plasticizer, and to constitute complexes with the pectin and xylans (Tan et al., 2012). A complex arabinoxylan pectin arabinogalactan protein1 (APAP1) formed by the covalent interaction between *A. thaliana* AGP hemi cellulosic and pectic polysaccharide, has been reported to play some structural role in the CW (Tan et al., 2013). However, it is hypothesized that AGP31 established non-covalent cohesion in networks residing within the CW and could be extracted from the CW polysaccharides. The AGPs showed prominence in the covalent linkages with the pectin and hemicelluloses to restructure an APAP1 complex (Tan et al., 2013).

The AGPs determine plant growth, cell division, necrosis, zygotic division, and embryo formation (Majewska-Sawka and Nothnagel, 2000). They also play a role in somatic embryogenesis during embryonic development of plants (Businge and Egertsdotter, 2014). In *Arabidopsis*, the AGPs were located in the basal part of the suspensor (Hu et al., 2006). In *Nicotiana*, the expression of AGPs during the embryo development stage was highly regulated (Geshi et al., 2013). Moreover, the potentiality of cellulose and pectin deposition in the CW was also hampered due to disruption in the AGPs functionality. Recent studies have focused on the structure biosynthesis and functionality of the AGPs enriched by a high percentage of sugars (Kitazawa et al., 2013). Thirteen genes encoding different types of AGPs with their roles are outlined in Table 2.

**Table 2:** **Arabinogalactan proteins genes in cell wall of *A. thaliana*^†^**.

**Locus**	**Gene ID**	**Biological process/Molecular function**	**Expressed in**	**Description**	**Some of the related references**
AT5G55730	*FLA1*	Root and shoot system development	Vascular leaf, Carpel, cauline leaf, collective leaf structure, guard cells, flower and inflorescence, cotyledon, flower, guard cell, hypocotyl, seed, root, and plant embryo	Fasciclin-like arabinogalactan protein 1	Sultana et al., 2015
AT2G45470	*AGP8*	–	Carpel, cauline leaf, leaf structure, guard cells, flower and inflorescence, cotyledon, flower, guard cell, hypocotyl, seed, root, and during different stages of plant embryo	Arabinogalactan protein 8	Macmillan et al., 2010
AT5G03170	*FLA11*	Plant-type secondary CW biogenesis	Carpel, cauline leaf, collective leaf structure, guard cells, flower, and inflorescence	Fasciclin-like arabinogalactan protein 11	Macmillan et al., 2010
AT5G14380	*AGP6*	Pollen tube growth and pollen tube viability	Carpel, leaf structure, flower, petal, plant embryo and inflorescence	Arabinogalactan protein 6	Jia et al., 2015
AT3G01700	*AGP11*	Pollen tube growth	Carpel, leaf, flower, embryo, pollen, stamen, and pedicel	Loss of AGP11 function results in unfertile pollen tube due to defective growth.	Costa et al., 2013
AT2G24450	*FLA3*	N-terminal protein myristoylation	Carpel, embryo, pollen, flower, and stamen	Fasciclin-like arabinogalactan protein 3	Johnson et al., 2011
AT2G04780	*FLA7*	–	Carpel, cotyledon, guard cell, inflorescence meristem, hypocotyl, shoot system, and leaf	Fasciclin-like arabinogalactan protein 7	Macmillan et al., 2010
AT5G44130	*FLA13*	–	Seed, root, leaf, flower, and embryo	Fasciclin-like arabinogalactan protein 13	Macmillan et al., 2010
AT5G60490	*FLA12*	Secondary cell wall biogenesis	Vascular root, leaf, flower parts and peduncle	Fasciclin-like arabinogalactan protein 12	Macmillan et al., 2010
AT5G10430	*AGP4*/ *JAGGER*	Synergid death	Stamen, petal, root, leaf system, seed, and hypocotyl	Arabinogalactan protein 4	Pereira et al., 2016
AT5G07830	*GUS2*	Extracellular matrix organization and uni-dimensional cell growth	Carpel, leaf, hypocotyl, root, seed, shoot, stem, and flower	A member of glycoside hydrolase family 79	Bayer et al., 2006
AT2G15390	*FUT4*	CW organization and CW biogenesis Alpha-(1,2)-fucosyltransferase activity	Carpel, leaf, hypocotyl flower, guard cell, stem, stamen, and whole plant	Fucosyltransferase 4	Tryfona et al., 2014
AT1G14080	*FUT6*	Fucosylation and cell wall biogenesis	Flower, root, stem	Fucosyltransferase 6	Liang et al., 2013

† *The information given in this table is based on TAIR database (Lamesch et al., 2012)*.

### Hybrid proline rich proteins (HyPRPs)

The HyPRPs determine cell-type-specific wall structure during developmental phases and contribute in defensive mechanisms during the pathogenic infection. When treated with fungal elicitor, physical damage, and pathogen infection, the HyPRPs were rapidly insolubilized in the CW (Francisco and Tierney, 1990). They are a group of structural proteins formulating covalent cross linkages between the constituents of the CW (Showalter, 1993). They are further categorized on the basis of deoxyribonucleic acid (DNA) sequence similarity, continuity of motifs and domain organization (Fowler et al., 1999). The HyPRPs belonged to classical protein families with well-defined sequence but little is known about their systematic functioning. The CW based molecular mechanisms involved in evolution; ontogeny and functioning were purely based on theoretical interests albeit its major role was defined as a physical support to the cell. The HyPRPs are classified on the bases of different domains, proline rich N terminal repetitive, and hydrophobic C terminal domains (Neto et al., 2013). Previous data has revealed that the expression level and stimuli for HyPRPs differ significantly owing to plant developmental stage and environmental conditions. The blast analysis of *Arabidopsis* genome sequence revealed the involvement of 28 HyPRPs gene loci in the CW functions (Chardon et al., 2004). All chromosomes contained HPRPs genes but the higher number had been reported on second chromosome. The expression pattern of these genes is partially conserved between closely related paralogous genes. The exact role and compartmentalization of PRPs is still not well-understood compared to some better-characterized families. The PRPs are least glycosylated proteins that are extremely basic with the demonstration of specific repetitive motifs. Although there is no expressive evidence, still it is predicted that PRPs were cross linked by covalent bond within the CW (Tan et al., 2003). With the help of online tools, we have reported four genes controlling different kinds of PRPs in CW of *A. thaliana*, which are involved in different types of molecular processes in different localities (Table 3).

**Table 3:** **Proline rich proteins genes in cell wall of *A. thaliana*^†^**.

**Locus**	**Gene ID**	**Biological process/Molecular function**	**Expressed in/Located in**	**Description**	**Some of the related references**
AT1G54970	*PRP1*	Trichoblast differentiation	Root, root hair cell, trichoblast/CW, extracellular region	Proline-rich protein 1	Bergonci et al., 2014
AT2G21140	*PRP2*	CW organization	Leaf, stems, flowers, inflorescence meristem, stem, guard cell, petal/CW, extracellular region	Proline-rich protein 2	Panjabi et al., 2008
AT3G62680	*PRP3*	Cellular responses to auxin stimulus and calcium ion starvation, and trichoblast differentiation	Root hair cell, trichoblast/CW, extracellular region	Proline-rich protein 3	Bergonci et al., 2014
AT4G38770	*PRP4*	Cysteine biosynthetic	Carpel, sepal, shoot apex, shoot system flower/CW, extracellular region	Proline-rich protein 4	Panjabi et al., 2008

† *The information given in this table is based on TAIR database (Lamesch et al., 2012)*.

## Glycine rich proteins (GRPs)

The GRPs located within the CW of vascular tissues are regulated during the developmental stages of plants (Ye et al., 1991) by forming a third major group of CW proteins. The manifestation of many GRPs takes place under environmental stresses like water deficiency, high light, ABA, and pathogenic infestations. Although, it is presumed that GRPs were a part of the plant defense system, their mechanism of action is not yet known. It is possible that GRPs presenting other functional domains were necessary to determine how protein activity get affected under different conditions (Mangeon et al., 2010).

In plants, GRPs related genes are regulated during developmental stages and their expression varies in plant tissues (Yan et al., 2015). In different genera of plants, the expression of such genes is controlled by biotic and abiotic stresses (Ahmad et al., 2014; Alghabari et al., 2015, 2016; Ihsan et al., 2015; Yan et al., 2015). In plants, the categorization of GRPs is based on the semi-repetitive glycine rich motifs (Sachetto-Martins et al., 2000). According to a report, the French bean PvGRP1.8, a class 1 GRP, located in un-lignified primary CW, played a structural role in protoxylem through the CW buttressing (Ryser et al., 2004). Reverse genetics approaches have fortified the concept of the involvement of GRPs gene from *Arabidopsis* in the deposition of the secondary CW and maintenance of proto-xylem structure (Yokoyama et al., 2007).

Undoubtedly, the diversified structure, intonation, expression, prototype, and subcellular localization of GRPs are strongly witnessed for their prominent role and functionality in plants (Sachetto-Martins et al., 2000). Observations have disclosed the involvement and modulation of GRPs in the defense mechanism under pathogenic attack (de Souza Cândido et al., 2011). The *NtClG1* gene was induced in turnip whose level was altered by tobacco virus, as shown by increased deposition of cellulose, which restricted the viral movement. This implicate structural roles of GRPs in conferring defense mechanisms in plants (Ueki and Citovsky, 2002). The GRPs constitute almost 70% glycine (Kar et al., 2012). Analysis done by immunocytochemistry has revealed their direct alliance with proto-xylem, where they play a prominent role in repair and stretching phase (Sachetto-Martins et al., 2000). It is perceived that the continuity of glycine rich domains produced beta pleated hydrophobic structure. An *in vitro* cross-linking experiment in the presence of peroxidases has explored the configuration of networks in Tyr-containing GRPs. Nonetheless, there is further need to do experimentation to generate data to support the characterization of intra and inter molecular networks involving GRPs.

The GRPs are presumed to be involved in promoting expression of genes in plants, exemplified through the involvement of RNA binding GRP gene *AtCSG2* and their regulation during flower development (Sachetto-Martins et al., 2000). The plants in which *AtCSG2* was silenced due to biotic or abiotic stress showed premature flowering with reduced stamens and abnormal embryo development (Fusaro et al., 2007). The GRPs yet isolated from plants are categorized as CW-GRPs, RNA-GRPs, and cytokeratin like GRPs (Sharma et al., 2012). Analysis conducted through bioinformatics tools has explored 12 genes controlling different types of GRPs in the CW of *A. thaliana*. These genes also perform salient molecular functions in different kinds of cells and plant parts (Table 4). The study has also revealed eight genes, which could not be categorized to any kind of CWPs and the functions of these genes are indicated in Table 5.

**Table 4:** **Glycine rich protein genes in cell wall of *A. thaliana*^†^**.

**Locus**	**Gene ID**	**Biological process/Molecular Function**	**Expression in tissues**	**Description**	**Some of related the references**
AT4G39260	*GPR8*	Alternative mRNA splicing, Innate immune response, responses to ABA, salt stress, cold/Nucleic acid and nucleotide binding	Carpel, hypocotyl, leaf, juvenile vascular leaf, flower, fruit, guard cell, plant cell, plant embryo, seed and seedling developmental stages, and whole plant	Glycine-rich RNA-binding protein 8	Leder et al., 2014
AT4G18280	–	–	Carpel, hypocotyl, leaf, juvenile vascular leaf, flower, fruit, guard cell, plant cell, plant embryo, shoot system, root and whole plant	Glycine-rich cell wall protein-related	Lan et al., 2007
AT3G23830	*GRP4*	Response to cold/RNA and DNA binding	Flower, guard cell, and cotyledon	Glycine-rich RNA-binding protein 4	Han et al., 2013
AT3G20470	*GRP5*	Response to ABA or salicylic acid stimulus, positive regulation of cell growth/CW structural constituent	Carpel, leaf, plant cell, Flower, fruit and leaf	Glycine-rich protein 5	Mangeon et al., 2010
AT5G07530	*GRP17*	Lipid storage, pollen hydration, sexual reproduction/lipid binding	Leaf, petal, pollen, flower, petal, sepal and stamen	Glycine-rich protein 17	Li-Beisson et al., 2010
AT5G07510	*GRP14*	Lipid storage, sexual reproduction/Nutrient reservoir activity	Collective leaf structure, flower, petal and sepal abundance it express in stems and with very low abundance it express in leaves	Glycine-rich protein 14	Li-Beisson et al., 2010
AT5G07520	*GRP18*	Lipid storage, sexual reproduction/Nutrient reservoir activity	Collective leaf structure, flower, guard cell, petal and sepal	Glycine-rich protein 18	Wellmer et al., 2004
AT5G07550	*GRP19*	Lipid storage, sexual reproduction/lipid binding	Carpel, cauline leaf, collective leaf structure, flower, petal, sepal and stamen	Glycine-rich protein 19	Peiffer et al., 2008
AT5G07540	*GRP16*	Lipid storage, sexual reproduction/lipid binding	Carpel, collective leaf structure, flower, petal, sepal and stamen	Glycine-rich protein 16	Ehlting et al., 2008
AT2G15340		–	Collective leaf structure, petal, flower, and pollen tube	Glycine-rich protein	Wang et al., 2008
AT1G48410	*AGO1*	Leaf proximal, distal pattern formation/miRNA and protein binding	Carpel, leaf lamina, and inflorescence	Glycine-rich protein	Micol-Ponce et al., 2014
AT3G15400	*ATA20*		Carpel, cauline leaf, collective leaf structure, flower, petal, sepal and guard cell	Anther 20. Encodes a protein with novel repeat sequences and a glycine-rich domain, which has a 53% identity to GRP1, a petunia glycine-rich CW protein	Xu et al., 2010

† *The information given in this table is based on TAIR database (Lamesch et al., 2012)*.

**Table 5:** **Multiple function proline based genes in cell wall of *A. thaliana*^†^**.

**Locus**	**Gene ID**	**Biological process/Molecular function**	**Expressed in**	**Description**	**Some of the related references**
AT5G14800	*P5C1*	Proline biosynthetic process/Pyrroline-5-carboxylate reductase activity	Carpel, flower, leaf, guard cell, seed, shoot apex, root, stamen, pollen tube cell, and cotyledon	Delta 1-pyrroline-5-carboxylate reductase	Funck et al., 2012
AT4G02330	*PME41*	CW modification/Pectin esterase activity	Carpel, flower, leaf, guard cell, seed, shoot apex, root, stamen, pollen tube cell and cotyledon	Encodes a pectin methyl esterase that is sensitive to chilling stress and brassinosteroid regulation	Qu et al., 2011
AT3G43270		CW modification, pectin catabolic process/Pectin esterase activity	Carpel, flower, leaf, guard cell, seed, shoot apex, root, stamen, pollen tube cell and cotyledon	Plant invertase/pectin methyl esterase inhibitor superfamily	Irshad et al., 2008
AT2G19760	*PFN1*/*PRF1*	Actin polymerization, cytoskeleton organization/Actin monomer binding	Carpel, flower, leaf, guard cell, seed, shoot apex, root, stamen, pollen tube cell and cotyledon	Profilin 1	Wang et al., 2009
AT3G25500	*AFH1*	Actin cytoskeleton organization/Protein binding	Carpel, flower, leaf, guard cell, seed, shoot apex, root, stamen, plant embryo, pollen tube cell and cotyledon	It is involved in signal-transduction cascade which results in rearrangement of the actin cytoskeleton	Rosero et al., 2016
AT2G02990	*RNS1*	Anthocyanin-containing compound biosynthetic process, RNA binding and endoribonuclease activity	Flower, guard cell, carpel, collective leaf structure, petal, and embryo	Ribonuclease 1 is involved in wound induced signaling independent of JA	Nishimura et al., 2014
AT5G14610		ATP binding	Carpel, flower, leaf, guard cell, seed, shoot apex, root, stamen, pollen tube cell and cotyledon	DEAD box RNA helicase family protein	Spencer et al., 2007
AT3G22070		–	Flower, guard cell, inflorescence meristem, root, seed, shoot apex	Proline-rich family like protein	–

† *The information given in this table is based on TAIR database (Lamesch et al., 2012)*.

## Gene co-expression functionality

In *A*. *thaliana*, 2,700 proteins express 6,200 highly reliable interactions. The interactive maps provided a dynamic approach in better understanding of the plant biological systems and a base for future crop improvement (Braun et al., 2011). The exploitation of co-expression networks in *Arabidopsis* provides a dimension to mine genes involved in the synthesis of CW and to unravel the structural hierarchy of CW in systematic progression (Obayashi and Kinoshita, 2009). Here, we have made queries through PubMed to understand the genes expression and co-expression in a wired way in the CW through different kinds of structural proteins. Through bioinformatics tools, 58 genes involved in proline based CW regulation were found. The established wired networks showed the genes co-expression, and interaction in structural components, biological processes and molecular functions by regulating the synthesis of proline based CW proteins. In regulation of biological processes, these genes have shown physical interaction 55.7%, co-expression 15.6%, genetic interaction 6.9%, shared proteins domains 3.6%, and co-localization 0.6% (Figure 3). However, in determining cellular components, these genes have revealed physical interaction 7.4%, co-expression 55.9%, genetic interaction 1.1%, shared proteins domains 2.9%, and co-localization 1.8%. Moreover, these proteins helped in signal transduction by regulating the molecular functions for which physical interaction 39.0%, co-expression 11.8%, genetic interaction 3.3%, shared proteins domains 14.6%, and co-localization 0.4% were calculated. Similarly, gene networking and interaction, based on biological process, molecular process and cellular component for protein rich protein extensins, GRPs, arabinogalactan proteins, and PRPs indicated the genes co-expression and interaction even when they were considered separately for a particular family of proteins (Figure 4). These wired networks as a whole, are the clusters of genes repertoire, interacting and co-expressing for different kinds of proteins present in the CW. However, out of this treasury of genes, we mined the genes interacting for a particular class of proteins. Therefore, in this complete wired network, we have showed genes, which are interacting for a specific family of proteins as dark (black) spots. However, light spots represented those genes whose interactions were not considered (Figure 4).

The dynamic signals of the environment, triggering the genetic regulatory network, fluctuate continuously in plant's life cycle with varying extent of predictability. Plants are sessile beings, so to cope with such fluctuating environment, they do not show behavioral adaptations but they respond by modulating their development and physiology (Leakey et al., 2009). The percent of expression of these mined genes was found variable (0–100%) in different anatomical parts of *A*. *thaliana*, as it was revealed by heat map constructed through META analysis (Figure 5A). Likewise, fluctuating manifestation of these genes was noticed for different developmental stages (Figure 5B).

A genome array map for 10 representative genes of *A. thaliana*, in 27 anatomical parts at different developmental stages (germination to senescence) was constructed that represented an alteration in gene expression level with changing developmental stages of the plant (Figure 6). A total of 93 proteins (Boudart et al., 2005) have been identified using proteomic and bioinformatics approaches. A comparison of rosette plants revealed the highly cell type specific involvement of CW proteomes regulated by multigenic families. The plant allocates 10% of their genome (~2,500 genes in *Arabidopsis*) to synthesize and rearrange the CW (McCann et al., 2007) indicated by the study of protein sequential annotation. Nevertheless, the number of mined genes in the CW of *A. thaliana* are limited but have been justified experimentally for their involvement in the CW formation. However, cis- regulatory DNA and proteins interact to synthesize the organized CW structure, assured by the synthesis and modifications of these basic constituents in a well-maintained and coordinated fashion, by specific enzymatic activities as revealed by transcriptional coordination of their related genes (Brown et al., 2005).

Xylan is involved in the development of secondary CW in plants and its synthesis network possesses a set of highly expressed genes. A comparative co-expression analysis between rice and *Arabidopsis* disclosed the absence of some gene families that were present in other species, indicating a clear difference in their CWs (Oikawa et al., 2010). Previous research has published numerous studies about gene interactions, gene expression and protein interactions, but now there is a major need to integrate this knowledge to understand the basic features of living organisms that are proceeding in an organized way. The different interactions including gene expression, gene interactions and protein interactions were used to assemble a biological network that has defined the basic principles to wire a network in a chronological way (Beyer et al., 2007).

Based upon available proteomics and genomics data sets of *A. thaliana*, geneMANIA can be used to estimate their gene function. To study co-expression and positive as well as negative correlation of gene expression profiles, we applied Plant Array Nets, which works equally good for *Oryza, Brassica*, and *Arabidopsis* (Warde-Farley et al., 2010) The immense knowledge as an outcome of genome sequencing paves the way to understand the working philosophy of genes in an integrated way on genome sequencing, expression analysis and protein interaction. The transcriptional coordination has been estimated using PCC. By using this method, co-expression relationships between many genes can be estimated, and visualized as a network in which nodes indicate genes whereas connection between nodes represents the transcriptional coordination of genes (Aoki et al., 2007). The PCC method sometimes becomes defective when some biological processes are strongly transcriptionally co-regulated, while other processes are not. In addition to this, a lower value of PCC results in excessively large gene clusters, possessing thousands of genes (Mao et al., 2009).

The PCC for 50 proline based CW regulating selected genes presented a positive and negative co-expression of +0.969 and −0.827, respectively (Figure 7). Hierarchical clustering of genes based on PCC indicated the co-expression of some genes with same intensity at particular developmental stage with altered expression level changing in the developmental stage (Figure 8). The genes that showed co-expression also represented a high degree of functional correlation. Co-expressions studies of genes can be used to identify other genes. For example, in cellulose synthesis, the co-expression approach can be used to identify the genes involved in the synthesis of hemicellulose (Cocuron et al., 2007). Many genes that are transcriptionally associated with the synthesis of the CW have been already studied (Ruprecht et al., 2011). The genetic redundancy needs mutant combinations or knocks down approaches that will focus upon several homologous genes to generate informative phenotypes. In addition to this, detailed comparative transcriptional studies are still required to mine candidate genes for the CW synthesis.

The co-expression analysis gives one possible caveat of “false positives,” which means some genes are co-expressed by chance rather than being functionally related. However, it has been reported that co-expression relationships are often conserved across species (Obayashi and Kinoshita, 2009). Hence, co-expression analysis across species can improve the reliance of co-expression based functional annotation.

Through computational methodologies, we have generated figures highlighting the expression of genes at different plant specific stages. The module-based predictions provide an approach to formulate hypothesis for functionally unknown genes (1,701) in *Arabidopsis* and other plant species. It also provides a new imminent into the conservation of co-expression and co-regulation (Heyndrickx et al., 2014). Through proteins architecture studies of the CW, we have identified several genes directly and indirectly involved in proteome manufacturing (Yang et al., 2011). In response to heat stress, *P5CR* launches its oxido-reductase activity by producing pyrroline-5-carboxylate reductase enzyme at the vicinity of the cytoplasm and CW. Under the conditions of biotic and abiotic stresses, the gene express itself in CW compartment by enhancing proline transport and increasing sensitivity against pathogenic stimuli (Bosch et al., 2011). Hence, modifications in CW proteins and proline transport are an indicator of regulation of genes expression under biotic stresses.

For co-expression studies, the bioinformatics tools have been focused on the model plant *Arabidopsis* by including the major bulk of publicly available microarray datasets. The candidate genes forming the foundation of the existing *A. thaliana* CW regulatory network, have been identified by gene expression profiling (Handakumbura and Hazen, 2012). Genes with similar functionality and overlapping effects, such as expression and regulation of floral developmental and defense related genes in response to biotic stress (Ahmad et al., 2013, 2014), can also be coordinated as indicated by global transcript analysis based upon publicly available microarray datasets. Certainly, through co-expression analysis in *A. thaliana*, many transcriptionally coordinated genes involved in the formation of CW proteins, cellulose, hemicelluloses and lignin have been identified. To facilitate this co-expression analysis, several helpful web based tools have been developed for the researchers to investigate transcriptional co-ordinations as well as to mine the candidate genes involved in the CW integrity. In addition, several tools paved the foundation to make comparative transcriptional analysis across many species, which will potentially increase predictive power about gene functionality.

## Author contributions

ZA, JNA, and MI came up with the ideas and reviewed all the literature; MI, SJNA, ZS, and HR took part in writing the manuscript. IA, JNA, and AMB reviewed, critically analyzed and edited the manuscript. All authors discussed and commented on the manuscript.

### Conflict of interest statement

The authors declare that the research was conducted in the absence of any commercial or financial relationships that could be construed as a potential conflict of interest.

## Abbreviations

CW: cell wall
CWPs: cell wall proteins
APG: adelaide protein group
At: *Arabidopsis thaliana*
APAP1: arabinoxylan pectin arabinogalactan protein1
FLA: fasciclin-like arabinogalactan
GUS2: glucuronidase
GP1: glycosyl phosphatidyl inositol
Hyp: hydroxyproline
LRKs: leucine rich repeats receptor like kinases
PTMs: post translational modifications
PRExts: proline rich extensin like proteins
PERKs: proline rich extensin like receptor kinases
LecRKs: lectin receptor kinases
P4Hs: prolyl 4-hydroxylases
P5CR: pyrroline-5-carboxylate reductase
RANBP: ran binding protein
PRPs: proline rich proteins
HRGPs: hydroxy-proline rich O-glycoproteins
AGPs: arabinogalactan proteins
CWAKs: cell wall associated kinases
ROS: reactive oxygen species
Asp: aspartate
Cys: cysteine
GRPs: glycine rich proteins
EXTs: extensions
FLAs: fasciclin-like AGPs
THRGP: threonine-rich hydroxyproline-rich glycoprotein
Tyr: tyrosine
DNA: deoxyribonucleic acid
